# Association between Urinary BPA Substitutes and Precocious Puberty among Girls: A Single-Exposure and Mixed Exposure Approach from a Chinese Case—Control Study

**DOI:** 10.3390/toxics11110905

**Published:** 2023-11-06

**Authors:** Francis Manyori Bigambo, Dandan Wang, Jian Sun, Xinliang Ding, Xiuzhu Li, Beibei Gao, Di Wu, Wei Gu, Mingzhi Zhang, Xu Wang

**Affiliations:** 1 Department of Endocrinology, Children’s Hospital of Nanjing Medical University, Nanjing 210008, China; francis.bigambo@yahoo.com (F.M.B.); wangdandanys@163.com (D.W.); guwei154@163.com (W.G.); 2 Department of Emergency, Pediatric Intensive Care Unit, Children’s Hospital of Nanjing Medical University, Nanjing 210008, China; malajian8492211@163.com; 3 Key Laboratory of Modern Toxicology of Ministry of Education, School of Public Health, Nanjing Medical University, Nanjing 211166, China; dingxinliang@njmu.edu.cn (X.D.); lxzwx888@163.com (X.L.); gaobb@njmu.edu.cn (B.G.); diwu@njmu.edu.cn (D.W.); 4 Wuxi Center for Disease Control and Prevention, The Affiliated Wuxi Center for Disease Control and Prevention, Nanjing Medical University, Wuxi 214023, China; 5 Research Base for Environment and Health in Wuxi, Chinese Center for Disease Control and Prevention, Wuxi 214023, China; 6 State Key Laboratory of Reproductive Medicine, Institute of Toxicology, School of Public Health, Nanjing Medical University, Nanjing 211166, China

**Keywords:** BPA substitutes, single exposure, mixed exposure, precocious puberty, case–control study

## Abstract

There is an argument that BPA substitutes may have the same or more deleterious health effects as BPA due to their structural similarity. This study explored the association between urinary BPA substitutes and precocious puberty among girls by including 120 girls with precocious puberty (cases) aged 2–10 years enrolled at Nanjing Children’s Hospital Department of Endocrinology in China between April 2021 to September 2021 and 145 healthy girls (controls) recruited from a primary school. Logistic regression was used to evaluate the effect of single exposures, and Bayesian kernel machine regression (BKMR) and quantile-based g-computation were used for the mixed effect. In the multivariate logistic regression, BPS (bisphenol S), TBBPA (tetrabromobisphenol A), and BPFL (bisphenol-FL) were significantly associated with increased risk of precocious puberty (odds ratio (OR) = 1.75, 95% confidence interval (CI): 1.13, 2.76, *p* = 0.014), (OR = 1.46, CI: 1.06, 2.05; *p* = 0.023), and (OR = 1.47, CI: 1.01, 2.18; *p* = 0.047), respectively. The BMKR and quantile-based g-computation models revealed consistent associations for single exposures and there was insufficient evidence for the associations of the mixed exposure of bisphenols with precocious puberty. In conclusion, BPA substitutes such as BPS, TBBPA, and BPFL may be associated with an increased risk of precocious puberty in girls.

## 1. Introduction

Bisphenol A (BPA) is a type of phenol among personal care products and plasticizing chemicals (PCPPCs). BPA is widely used in the industry for making polycarbonate plastics and epoxy resins [1]. It is estimated that 10 million tons of BPA could have been produced in 2022 [2] and 27 million plastic products containing BPA [3] are produced yearly globally, with the United States (U.S.) being the major producer. The routes of exposure to BPA in humans include ingestion via diet, inhalation of contaminated dust, and transdermal via the use of thermal paper or toys for children [4]. 

BPA is categorized as an endocrine-disrupting chemical (EDC) because of its ability to bind to estrogen and androgen receptor signaling pathways [5,6]. Epidemiological studies have linked BPA with precocious puberty including later pubarche and menarche among normal-weight girls [7], premature thelarche in girls [8,9], and increased idiopathic central precocious puberty (ICPP) in girls [10]. 

Because of the substantiated evidence of the deleterious health effects of BPA, numerous countries around the world have prohibited the use of BPA in some consumer products. The European Union has prohibited the use of BPA in infant feeding bottles and baby toys [11,12], and the U.S. Food and Drug Administration has prohibited the use of BPA in baby bottles and cups and coating packaging of infant formula [13,14]. Other countries that banned BPA use in some consumer products include China and Malaysia [15]. Given that, BPA analogs were introduced as BPA substitutes, including bisphenol S (BPS), bisphenol AF (BPAF), bisphenol AP (BPAP), bisphenol B (BPB), 4,4-(9-fluorenylidene) diphenol (bisphenol-FL) [BPFL], tetrabromobisphenol A (TBBPA), and tetrabromobisphenol S (TBBPS). 

Indeed, the structure of BPA substitutes is almost similar to BPA with exception to the side group introduced. This has raised health and safety concerns that BPA substitutes may have the same or more deleterious health effects than their parent BPA [16,17]. Evidence from in vivo and in vitro studies has substantiated that BPA substitutes such as BPS have estrogenic, anti-estrogenic, androgenic, and anti-androgenic potencies like BPA [1,16]. In other studies, BPS was observed to be an endocrine disruptor that alters the levels of plasma steroid hormones and gonadotropins in rats [18,19]. Shi and colleagues have shown that BPS exposure caused advanced precocious puberty onset in female mice [20]. Furthermore, BPFL has been linked with early development in zebrafish [21]. In humans, BPF and BPS have been observed to significantly associate with reproductive hormones in children, similar to BPA [17]. Little is known about the association of BPA substitutes with pubertal timing in children. Therefore, our study aimed to explore the association between urinary BPA substitutes including BPS, BPAF, BPAP, BPB, BPFL, TBBPA, and TBBPS with precocious puberty among girls in Nanjing China.

## 2. Materials and Methods

### 2.1. Population and Study Design

We performed a case–control study involving 121 girls aged 2–10 years diagnosed to have precocious puberty (cases) enrolled at Nanjing Children’s Hospital Department of Endocrinology in China between April 2021 and September 2021 and 149 healthy girls (controls) recruited from a primary school. We matched case and control groups at recruitment by age difference up to 5 years, as reported elsewhere [22]. Precocious puberty was diagnosed by a pediatrician in children who were residents of Nanjing City in Jiangsu province China. Bisphenols were detected in 120 cases and 145 controls, as 1 case and 4 controls did not provide urine samples for bisphenols detection and were excluded as shown in Figure S1. 

In the current study, we use data that cannot portray an individual identity. The Institution Review Board of the Children’s Hospital of Nanjing Medical University approved the study (Reference number 202101014-1) and participants’ written informed consent was obtained from their guardians. 

### 2.2. General Information and Physical Examination

We used structured questionnaires with five sections during the face-to-face interview to gather information about the subjects. (1) General information about children including child age, race, and residence, as well as mother parity, mode of delivery, guardians’ education level, and mother and father histories of early puberty. (2) Physical examination of parents and their children. The heights and weights of mothers and fathers were self-reported by guardians, the heights and weights of the cases were extracted from the hospital record, and the heights and weights of the controls were measured at recruitment. (3) The child’s assessed diet includes feeding methods after birth, frequencies of eating fried food and dessert, as well as drinking soft drinks and juice. (4) Sleeping patterns include averaged sleep hours per 24 h and child snores. (5) Parenting styles such as child time on screening devices per day, time spent in outdoor activities, the intensity of outdoor activities, and secondhand smoke exposure.

### 2.3. Precocious Puberty Measurement

A pediatrician diagnosed precocious puberty based on the following criteria. (1) The occurrence of secondary sexual characteristics prior to eight years old in girls. Breast buds were the first manifestation. (2) There was a linear growth acceleration over the reference age group. (3) There was an advancement of bone age to actual age by 1 year or greater. (4) The image of B ultrasound revealed an increased volume of the uterus and ovary and the appearance of numerous ovarian follicles of diameter greater than 4 milliliters. (5) Active hypothalamus pituitary—gonadal axis and the luteinizing hormone (LH) peak ≥5.0 U/L and the ratio of LH and follicle-stimulating hormone (FSH) peak >0.6 in the luteinizing hormone-releasing hormone (LHRH) stimulation experiment. Cases with secondary central precocity factors such as infection and congenital dysplasia, central nervous system problems, radiotherapy, trauma, chemotherapy, and those postoperative were excluded. Additionally, cases with conditions such as McCune—Albright syndrome and adrenal hyperplasia were also excluded along with those with inborn hypothyroidism. For the control group, the controls were healthy girls not meeting the criteria for precocious puberty diagnosis. 

### 2.4. Measurement of Bisphenols

Morning urine specimens of the cases and controls were collected in urine collection cups and stored frozen in polypropylene containers at −20 °C for the analyses of bisphenols, including bisphenol A (BPA), bisphenol S (BPS), bisphenol AF (BPAF), bisphenol AP (BPAP), bisphenol B (BPB), bisphenol-FL (BPFL), tetrabromobisphenol A (TBBPA), and tetrabromobisphenol S (TBBPS), as detailed in the previous study [23]. At a glance, urine samples were spiked by 20 µL of mixed standards (MS) of methanol and 20 µL of mixed internal standards (IS) of methanol before extraction to ensure accurate and precise quantification of the analytes in the samples. Then, 200 µL solution containing buffers and deconjugated enzyme containing 50 units of β-glucuronidase (35 mL buffers + 262.5 µL β-glucuronidase) was added to the samples at pH 5. The samples were mixed thoroughly by vortexing for 10 s. Thereafter, 100 µL of urine was added to the samples, and the samples were mixed by vortexing for 10 sec and then incubated at 37 °C for 120 min. For the blank sample, 100 µL of ultrapure water was added as a substitute for urine. After incubation, 400 µL ethyl acetate was added to the samples. The samples were mixed by vortexing for 15 min, and ultrasonic inspection was performed for 20 min. Then, 50 mg MgSO_4_ was added to the samples to stop the activity of the enzyme. The samples were mixed by vortexing for 30 s and separated by centrifuging at 10,000 relative centrifugal force (RCF) for 15 min for extraction. The supernatants were concentrated gently under vacuum drying at 35 °C until approximately 50 µL was obtained. After that, the samples were reconstituted by 50 µL of a methanol–water mixture (*v*:*v* = 1:1) and mixed thoroughly by vortexing for 20 s. The samples were transferred into a 0.2 um filter and centrifuged at 10,000 rpm for 1 min. Filtered samples were transferred into Ultra-High-Performance Liquid Chromatography-Tandem Quadrupole Linear Ion Trap Mass Spectrometry (UHPLC- QTRAP) for quantification. The limit of detections (LODs) for BPA, BPS, BPAF, BPAP, BPB, BPFL, TBBPA, and TBBPS were 0.05 ng/mL, 0.05 ng/mL, 0.01 ng/mL, 0.05 ng/mL, 0.05 ng/mL, 0.05 ng/mL, 0.08 ng/mL, and 0.01 ng/mL, respectively. The data below LODs were imputed by taking LOD over the square root of 2.

### 2.5. Statistical Analysis

Categorical variables were compared using the chi-square (ꭓ2) test or Fisher’s test and the results were presented as participant numbers (N) and percentages (%). Continuous variables were compared using a *t*-test and the results were presented as mean (standard deviation, SD), as the variables were normally distributed in the histogram graph. Since the distributions of the bisphenols were right-skewed, base-10 logarithm transformation was introduced to improve normality. Pearson’s correlation coefficients test was conducted to evaluate the bivariate correlation between the bisphenols.

#### 2.5.1. Unconditional Logistic Regression 

Firstly, we used unconditional logistic regression to evaluate the association of bisphenols with the cases and controls. The results were presented as odds ratio (OR) and 95% confidence interval (CI). Covariates that revealed statistically significant differences between the cases and controls were entered into the directed acyclic graph to select the minimal sufficient variables to be adjusted in the models (Figure S2). The missing values in the covariates (1.5–10.9%) were imputed by random forest in the missForest in R.

#### 2.5.2. Bayesian Kernel Machine Regression (BKMR)

Secondly, we applied BKMR to evaluate the association between mixed bisphenols and precocious puberty. BKMR is a supervised non-parametric approach that assesses the association of the mixture with the outcome. It uses a kernel function to stipulate the unidentified exposure–response association, permits nonlinear and interaction relations between chemicals, and incorporates the covariates [24]. In the present study, we implemented a hierarchical variable selection method to appraise the posterior inclusion probabilities (PIPs) for group inclusion and conditional in BKMR. The model was fitted with the Markov Chain Monte Carlo (MCMC) algorithm for 10,000 iterations. Because PIP does not show the direction of associations, we evaluated the direction of associations of single exposures to bisphenols and precocious puberty when other bisphenols were set at their median value. We assessed the effect of the mixed exposure of bisphenols on precocious puberty when bisphenols were set at different percentiles (25th, 30th, 35th, 40th, 45th, 50th, 55th, 60th, 65th, 70th, or 75th) and compared with their 50th percentile (median). Then, we evaluated the bivariate interactions between bisphenols when a single exposure-outcome function of the mixed bisphenols by second exposure of the mixed bisphenols was set at different percentiles (10th, 50th, and 90th) and compared it with the remained bisphenols set at their median. The interaction was confirmed when a single exposure of bisphenols was set at percentiles 75 to 25th and compared to other bisphenols set from percentiles 25, 50, and 75th. We used the bkmr package (version 0.2.0) for analysis.

#### 2.5.3. Quantile-Based g-Computation

Finally, we performed quantile-based g-computation to evaluate the mixture effect of bisphenols on precocious puberty using the qgcomp.glm.noboot function. Quantile-based g-computation assesses the effect of the mixture on the outcomes and identifies the contributions of each component to the outcome by giving weight to each component in either a positive or negative direction [25]. The qgcomp package was used for analysis.

We used Stata software (version 17) for descriptive analysis and the rest of the analyses were performed in R (version 4.0.2). The level of significance was set at *p* < 0.05 (Two-sided).

## 3. Results

### 3.1. Study Population Characteristics

Table 1 shows the characteristics of the study participants of 120 cases and 145 controls. The mean age of the cases and controls was 7.73 (1.09) and 6.96 years, respectively. The majority of the participants (74.79% cases and 90.70% controls) were living in the city. The guardian education of most of the participants (29.82% cases and 34.68% controls) was university level and most had 1 child (para 1) (89.47% cases and 72.27 controls). The mean BMI of the participants’ mothers was 22.63 (3.90) kg/m^2^ for cases and 24.03 (8.02) kg/m^2^ for controls, and the mean sleep duration of children per day was 8.89 h for cases and 9.15 h for controls. The average time spent in outdoor activities of most of the children was often but less than 1 h.

### 3.2. Urinary Concentration of Bisphenols

Detection rates and urinary concentration of bisphenols between the cases and controls are presented in Table 2. The highest detection rates of more than 85% were detected in BPA (99.31% cases and 98.33 controls), BPS (95% cases and 99.17% controls), BPB (100% cases and 99.17% controls), and BPFL (89.66 cases and 97.50% controls). The detection rates of more than 49% were found in TBBPA (73.10% cases and 85.00% controls), BPAF (49.66% cases and 58.33% controls), and BPAP (59.31% cases and 59.17% controls). TBBPS had the lowest detection rate (11.03% cases and 11.67% controls) and was not included in the final analysis. 

We assessed the correlation coefficients (r) among eight bisphenols separately for cases and controls; statistical significance was set at *p* < 0.05 as shown in Figure S3. In the cases, all seven BPA substitutes were statistically significantly correlated while BPA was only correlated with BPB (r = 0.18). The highest and lowest coefficient correlations among bisphenols were 0.06 and 0.8, respectively. In the controls, BPA was not statistically significantly correlated with any of the BPA substitutes, while among the BPA substitutes, BPAF vs. TBBPS (r = 0.15) and BPAP vs. TBBPS (r = 0.16) were not statistically significantly correlated. The highest and lowest coefficient correlations among bisphenols were 0.03 and 0.80, respectively. 

### 3.3. Logistic Regressions to Evaluate the Association between Bisphenol Exposures and Precocious Puberty 

The associations between bisphenol exposure and precocious puberty are presented in Table 3. In the multivariate logistic regression, BPS (OR = 1.75, 95% CI: 1.13, 2.76; *p* = 0.014), TBBPA (OR = 1.46, 95% CI: 1.06, 2.05; *p* = 0.023), and BPFL (OR = 1.47, 95% CI: 1.01, 2.18; *p* = 0.047) were statistically significantly associated with increased risk of precocious puberty. This association was also present in the univariate logistic regression. Moreover, in the univariate logistic regression, BPA was statistically significantly associated with decreased risk of precocious puberty (OR = 0.39, 95% CI: 0.19, 0.80; *p* = 0.012). However, this association was not present in the multivariate logistic regression. The rest of the BPA substitutes did not show any significant associations.

### 3.4. BKMR to Evaluate the Association of the Mixed Exposure of Bisphenols with Precocious Puberty

In the BKMR hierarchical variable selection method, bisphenols were categorized into two groups based on their correlations assuming that all chemicals follow the same biological mechanism. Group 1 contains BPA and BPB, and Group 2 contains BPS, BPAF, BPAP, TBBPA, and BPFL. A threshold value of 0.5 was regarded to be significant. The posterior inclusion probabilities (PIPs) for group inclusion and conditional in BKMR are presented in Table S1. The group PIPs for both groups 1 and 2 were 0.88 and 0.74, respectively. BPA in group 1 and TBBPA in group 2 had the highest conditional PIPs, (0.90) and (0.53), respectively, indicating that these two chemicals had more impact on the model in their respective groups. 

**Figure 1:**
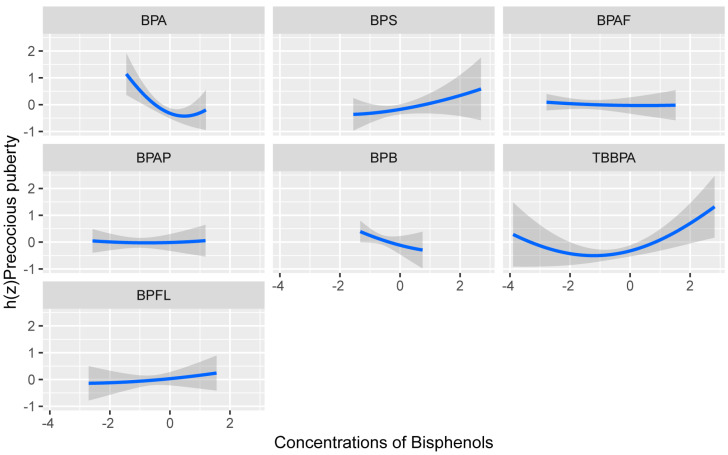
The risk of precocious puberty (95% credible interval) at each exposure of bisphenols by comparing the median of each bisphenol, while all other bisphenols were set at their median values. The Bayesian kernel machine regression was used to fit the models, adjusted for child age, child resident, child body mass index (BMI), guardian education, parity, mother’s BMI, sleep duration, and time spent in outdoor activities.

We evaluated the direction of associations of a single exposure to bisphenols and precocious puberty, by comparing the median of each bisphenol, while all other bisphenols were set at their median values as shown in Figure 1. We found that the direction of associations of BPS, TBBPA, and BPFL was consistent with that observed in the multivariate logistic regression. 

**Figure 2:**
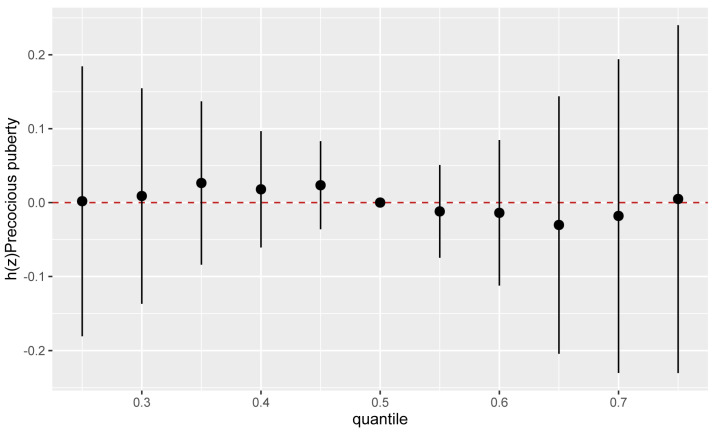
The overall risk of precocious puberty (95% credible interval) due to mixed exposure to bisphenols when all bisphenols were set at different percentiles and compared to their median (50th percentiles). The Bayesian kernel machine regression was used to fit the models, adjusted for child age, child resident, child body mass index (BMI), guardian education, parity, mother’s BMI, sleep duration, and time spent in outdoor activities.

**Figure 3:**
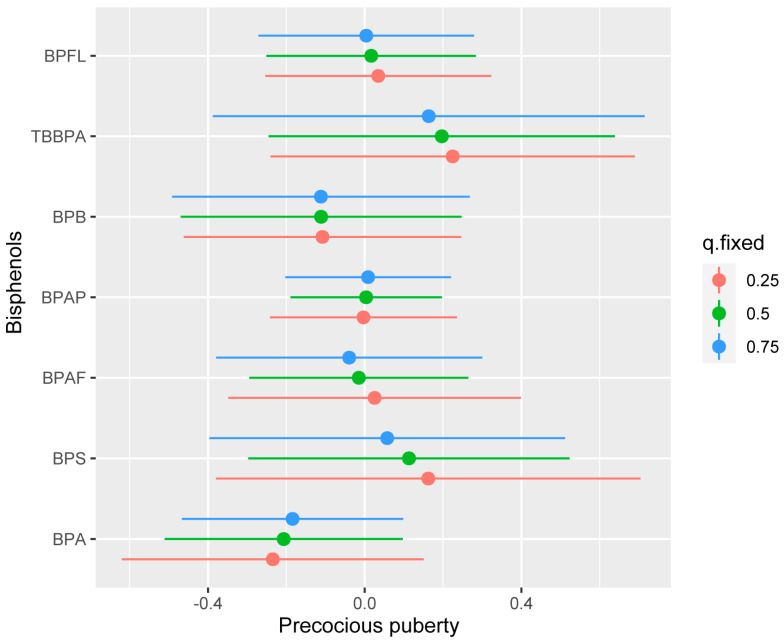
The risk of a single exposure to bisphenols (95% credible interval) on precocious puberty when a single exposure of bisphenols was set at percentiles 75 to 25th and compared to other bisphenols set from percentiles 25, 50, and 75th. The Bayesian kernel machine regression was used to fit the models, adjusted for child age, child resident, child body mass index (BMI), guardian education, parity, mother’s BMI, sleep duration, and time spent in outdoor activities.

Insufficient evidence was found for the associations of the mixed exposure of 7 bisphenols with precocious puberty when all bisphenols were set at different percentiles and compared to their median (Figure 2). Moreover, there were no significant interactions between bisphenols when a single exposure of bisphenols was set at percentiles from 75 to 25th and compared to other bisphenols set at percentiles 25, 50, and 75th (Figure 3). This was also confirmed when the single exposure–outcome function of the mixed bisphenols by second exposure of the mixed bisphenols was set at percentiles 10th, 50th, and 90th percentiles, and compared with the remaining bisphenols set at their median (Figure S4). 

### 3.5. Quantile-Based g-Computation to Evaluate the Association of the Mixed Exposure of Bisphenols with Precocious Puberty

**Figure 4:**
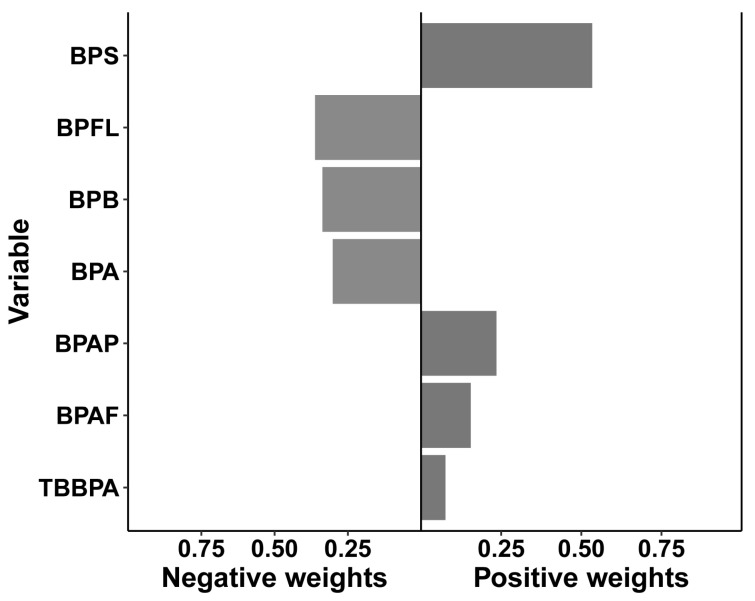
Relative contributions of seven bisphenols to precocious puberty. Quantile-based g-computation (QGC) was used to fit the model, adjusted for child age, child resident, child body mass index (BMI), guardian education, parity, mother’s BMI, sleep duration, and time spent in outdoor activities.

In the quantile-based g-computation model, the mixed exposure to seven bisphenols was not associated with precocious puberty (model 3, OR = 0.12, 95% CI: −0.30, 0.53; *p* = 0.578) (Table S2) similar to the BKMR result. BPS (weight = 0.53), BPAF (weight = 0.16), BPAP (weight = 0.24), and TBBPA (weight = 0.08) contributed positively to precocious puberty while BPA (weight = 0.30), BPB (weight = 0.34), and BPFL contributed negatively (Figure 4, Table S2). The relative contributions of BPS and TBBPA to precocious puberty were consistent with the results from logistic regression and BKMR models.

## 4. Discussion

This study aimed to investigate the association between urinary BPA substitutes and precocious puberty. Out of seven urinary bisphenols with a detection rate greater than 49%, only three BPA substitutes including BPS, TBBPA, and BPFL were related to a higher risk of precocious puberty. The BMKR and quantile-based g-computation models revealed consistent associations for certain single exposures and there was insufficient evidence for the associations of the mixed exposure of bisphenols with precocious puberty. Furthermore, there were no detected interactions between bisphenols. 

Epidemiological studies have linked BPA with later pubarche and menarche among normal-weight girls [7], premature thelarche in girls [8,9], and increased ICPP in girls [10]. In the present study, insufficient evidence was found to associate BPA with precocious puberty. A similar finding was also found in previous studies [26,27,28,29]. 

Although, we did not find an association between BPA and precocious puberty among girls, a large body of literature has documented substantiated evidence on the deleterious health effects of BPA, leading to the prohibition of the use of BPA in some consumer products across countries around the world [11,12,13,14,15]. This action accelerated the introduction of BPA analogs as BPA substitutes. Some studies have explored the effect of BPA substitutes in humans [17,30,31]. None of these studies assessed the effects of precocious puberty in girls. To the best of our knowledge, this is the first study to investigate the association between urinary BPA substitutes and precocious puberty among girls.

A study has documented reproductive and neuroendocrine function effects such as the secretion of E2, LH, and FSH in zebrafish embryos exposed to BPS [32]. BPS was related to delayed vagina opening in female rats [18]. Postnatal BPS was also related to serum TT in female mice [33], while prenatal BPS exposure was related to serum estradiol 17β in female mice [34]. Another study by Shi and colleagues revealed a positive association between BPS and advanced precocious puberty in female mice [20] similar to our findings. It is plausible that BPS may hinder the knockdown of germ cell nests in ovary development, disturb estrous cycles, and advance puberty onset [20]. Furthermore, BPS may directly affect the production of GnRH from the brain or directly inhibit pituitary production of gonadotropin hormones [35]. In humans, BPS was related to elevated SHBG and decreased TT in U.S males [31] as well as reduced serum E2, and SHBG in Chinese men [30]. The discrepancies in the results may be caused by differences in species, time of exposure, exposure doses, sex, and sample size among studies. 

Moreover, previous animal studies have documented TBBPA to inhibit the production of TT [36], and also exert estrogenic [37] or anti-estrogenic characteristics [38]. Changes in reproductive hormones such as E2, FSH, LH, and TT were also observed elsewhere [39]. In our study, we found that TBBPA was associated with precocious puberty among girls. This could be through its ability to increase the secretion of gonadotropin hormone [40,41], which in turn leads to precocious puberty onset.

Furthermore, BPFL has been linked to the disruption of oocyte maturation in mice [42], exerts anti-estrogenic characteristics, and causes reproductive and developmental toxicity in mice [43]. In our study, we found that BPFL was related to an increased risk of precocious puberty in girls. It could be plausible that BPFL triggers gonadal hypersecretion of reproductive hormones, which in turn leads to precocious puberty.

In the current study, BPAP, and BPB were not related to precocious puberty among girls. However, in a previous study, BPB was proposed to exert reproductive and neuroendocrine system effects such as secretion of E2, LH, and FSH in zebrafish embryos [32]. More research is required to investigate the toxicity effects of these BPA substitutes on pubertal timing due to data scarcity. 

In the present study, we can suggest that exposure to BPA substitutes such as BPS, TBBPA, and BPFL may be related to precocious puberty among girls through their ability to act on the reproductive neuroendocrine system. Generally, normal or abnormal pubertal development occurs through the action of reproductive hormones produced by the hypothalamus and anterior pituitary gland [35]. These BPA substitutes may directly affect the production of GnRH from the hypothalamus or directly inhibit pituitary production of gonadotropin hormones [35]. Since the GnRH performs an important role in regulating estrogen receptors, which are involved in puberty onset and ovulation, any abnormality changes in hormones from the toxicity effects of BPA substitutes may lead to abnormal pubertal development, including precocious puberty [44]. 

This study has numerous strengths. First, we recruited 120 girls diagnosed to have precious puberty as cases and 145 normal health girls as controls; no statistically significant difference in age was observed between the cases and controls included in our study. Second, we were able to assess the single-exposure effect of BPA substitutes on precocious puberty along with the mixed effect of BPA substitutes by employing the BKMR and quantile-based g-computation approaches. The BKMR approach is a recent method for dealing with environmental mixture exposure due to its ability to detect the non-linearity or interaction between the exposures and determine the overall effect of the mixture. Quantile-based g-computation assesses the effect of the mixture on the outcomes and identifies the contributions of each component to the outcome by giving weight to each component in either a positive or negative direction. Lastly, the current study contributes a body of evidence to the literature that BPA substitutes such as BPS, BPFL, and TBBPA may be related to an increased risk of precocious puberty among girls. Since there is not enough evidence from human studies on the effect of BPA substitutes on precocious puberty in children, more research on humans should be conducted to draw a concrete conclusion about the effect of these chemicals, which are considered to work safely.

This study is not without its limitations. First, the participant’s general information such as parent physical examination(mother height and weight, father height and weight), child diet, sleep hours, outdoor activities, and use of screening devices were gathered through interview questionnaires with the guardians—this could lead to recall bias. Second, in our study participants’ ages ranged from 2 to 10 years old, indicating that some of the participants’ ages were older than the corresponding diagnostic criteria at the time of diagnosis, meaning these girls attended the hospital long after the appearance of secondary sexual characteristics, although there was no significant difference in age between the cases and controls in the descriptive analysis. Reflecting on the criteria for diagnosis of precocious puberty, this age difference may have been an issue in the inclusion criteria. Third, the half-life of BPA substitutes in the body are approximated to be less than 1 or 2 days depending on the type of substitute used. Measurement of BPA substitutes with their short half-lives in urine samples collected at a single point of time after diagnosis of precocious puberty cannot characterize long-term exposure to BPA substitutes prior to the onset of precocious puberty. However, in our study, we assumed that the level of exposure to BPA substitutes among the subjects was fairly stable before or after precocious puberty diagnosis due to continuous use habits of personal care and consumer products containing BPA substitutes [27]. Fourth, our study is a single-center case–control study; the results cannot be generalized to the whole population in China. A multicenter case–control study is required to reach a such conclusion. However, our study revealed that BPA substitutes are related to an increased risk of precocious puberty among girls in Nanjing China. 

## 5. Conclusions

Our results suggest that BPA substitutes, specifically BPS, BPFL, and TBBPA may be related to an increased risk of precocious puberty among girls. This result demonstrated the possible health effects of BPA substitutes, chemicals that are currently considered to work safely. Since there are scarce data on the effect of BPA substitutes on pubertal development in children, researchers should pay more attention to the health and safety concerns of BPA substitutes on pubertal timing in humans to protect public health, particularly that of younger children.

## Figures and Tables

**Table 1:** Characteristics of the study participants.

Variables	Cases (N = 120)	Control (N = 145)	*p* Value
Age Mean (SD)	7.73 (1.09)	6.96 (0.53)	1.000
Race			
Han	117 (97.50)	129 (98.47)	0.459 *
Others	3 (2.50)	2 (1.53)	
Missing	0	14	
Residential area			0.002
City	89 (74.79)	117 (90.70)	
Township	21 (17.65)	6 (4.65)	
Countryside	9 (7.56)	6 (4.65)	
Missing	1	16	
Guardian education			0.007 *
Elementary school or below	2 (1.75)	0 (0.00)	
Junior high school	31 (27.19)	18 (14.52)	
High school/technical school	26 (22.81)	21 (16.94)	
College/vocational college	21 (18.42)	42 (33.87)	
University	34 (29.82)	43 (34.68)	
Missing	6	21	
Para			0.001
1 child	102 (89.47)	86 (72.27)	
≥2 children	12 (10.53)	33 (27.73)	
Missing	6	26	
Delivery method			0.877
Natural birth	62 (51.67)	72 (50.70)	
Cesarean section	58 (48.33)	70 (49.30)	
Missing	0	3	
Preterm birth			0.212
Yes	10 (8.40)	6 (4.55)	
No	109 (91.63)	126 (95.45)	
Missing	1	13	
Child BMI (kg/m^2^)	17.84 (10.39)	16.50 (3.64)	0.917
Mother BMI (kg/m^2^)	22.63 (3.90)	24.03 (8.02)	0.041
Father BMI (kg/m^2^)	25.90 (8.74)	26.63 (7.32)	0.235
Missing	1		
Mother’s early history of puberty			
Yes	2 (1.67)	1 (1.08)	1.000 *
No	118 (98.33)	92 (98.92)	
Missing	0	52	
Father’s early history of puberty			0.105 *
Yes	3 (2.50)	0 (0.00)	
No	117 (97.50)	133 (100.00)	
Missing	0	12	
Feeding method after birth			0.440
Breastfeeding	55 (46.61)	59 (41.55)	
Formula	19 (16.10)	19 (13.38)	
Mixed feeding	44 (37.29)	64 (45.07)	
Missing	2	3	
Fried food			0.670
Yes	84 (71.19)	81 (68.64)	
No	34 (28.81)	37 (31.36)	
Missing	2	27	
Desserts			0.441
Yes	107 (92.24)	118 (89.39)	
No	9 (7.76)	14 (10.61)	
Missing	4	13	
Soft drinks			0.244
Yes	60 (50.85)	47 (43.12)	
No	58 (49.15)	62 (56.88)	
Missing	2	36	
Child sleeping hours (24 h)	8.89 (1.46)	9.15 (0.67)	0.028
Missing	4	2	
Child Snore			0.611 *
Never	37 (31.62)	56 (39.44)	
Only on cold or allergy	32 (27.35)	33 (23.24)	
Sometimes	45 (38.46)	49 (34.51)	
Always	3 (2.56)	4 (2.82)	
Missing	3	3	
Time on screening devices per day			0.716 *
Never	17 (14.66)	27 (18.88)	
<30 min	49 (42.24)	65 (45.45)	
30–60 min	26 (22.41)	28 (19.58)	
1–2 h	22 (18.97)	20 (13.99)	
>2 h	2 (1.72)	3 (2.10)	
Missing	4	2	
Time spent in outdoor activities			0.011 *
None	1 (0.85)	1 (0.70)	
Rarely	24 (20.51)	20 (14.08)	
Often but less than 1 h	58 (49.57)	51 (35.92)	
1–3 h	28 (23.93)	62 (43.66)	
More than 3 h	6 (5.13)	8 (5.63)	
Missing	3	3	
The intensity of outdoor activities			0.484
High	32 (27.59)	31 (21.83)	
Moderate	67 (57.76)	92 (64.79)	
Low	17 (14.66)	19 (13.38)	
Missing	4	3	
Secondhand smoke exposure			0.766
None	63 (53.85)	69 (47.92)	
Several times a month but less than once a week	20 (17.09)	31 (21.53)	
Several times a week but less than once a day	17 (14.53)	22 (15.28)	
Almost everyday	17 (14.53)	22 (15.28)	
Missing	3	1	

Abbreviations: SD, standard deviation; BMI, body mass index. Chi-square (ꭓ2)/Fisher’s test and *t*-test were between the cases and controls. * Indicate Fisher’s test. *p* value < 0.05.

**Table 2:** Distribution of Urinary bisphenols concentration among the cases (N = 120) and controls (N = 145).

Bisphenols (ng/mL)	Group	LOD (ng/mL)	Detection Rate (%)	GM	Percentiles						
					Min	5	25	50	75	95	Max
BPA	Cases	0.05	99.31	0.66	0.04	0.24	0.39	0.60	1.08	2.67	8.60
	Control	0.05	98.33	0.86	0.04	0.32	0.56	0.82	1.27	3.20	15.75
BPS	Cases	0.05	95.86	1.32	0.04	0.16	0.48	1.07	2.76	26.83	497.5
	Control	0.05	99.17	0.76	0.03	0.07	0.20	0.69	1.94	18.29	55.58
BPAF	Cases	0.01	49.66	0.08	<0.01	0.01	0.01	0.03	0.90	10.28	18.54
	Control	0.01	58.33	0.06	<0.01	0.01	0.01	0.01	0.70	7.04	32.77
BPAP	Cases	0.05	59.31	0.14	0.01	0.02	0.04	0.11	0.47	2.35	4.99
	Control	0.05	59.17	0.11	<0.01	15.28	0.01	0.04	0.08	0.39	1.85
BPB	Cases	0.05	100.00	0.33	0.05	0.09	0.18	0.28	0.53	2.21	3.89
	Control	0.05	99.17	0.32	0.06	5.65	0.09	0.15	0.30	0.60	1.78
TBBPA	Cases	0.05	73.10	0.48	<0.01	0.02	0.14	0.43	2.14	15.90	653.00
	Control	0.05	85.00	0.27	<0.01	38.10	<0.01	0.04	0.34	1.24	10.97
TBBPS	Cases	0.08	11.03	0.07	0.01	0.06	0.06	0.06	0.06	0.40	80.79
	Control	0.08	11.67	0.07	<0.01	21.32	0.06	0.06	0.06	0.06	0.56
BPFL	Cases	0.01	89.66	0.39	0.01	0.02	0.12	0.34	1.44	8.00	16.17
	Control	0.01	97.50	0.25	<0.01	0.01	0.05	0.30	1.02	4.39	35.57

Abbreviations: LOD, limit of detection; GM, geometric mean; Min, minimum; Max, maximum.

**Table 3:** Association between bisphenols and precocious puberty in girls (N = 120 cases and 145 controls).

Bisphenols	Precocious Puberty			
	Univariate Logistic Regression		Multivariate Logistic Regression	
	OR (95% CI)	*p* Value	OR (95% CI)	*p* Value
BPA	0.39 (0.19, 0.80)	0.012	0.44 (0.17, 1.10)	0.082
BPS	1.59 (1.13, 2.27)	0.008	1.75 (1.13, 2.76)	0.014
BPAF	1.10 (0.89, 1.35)	0.385	1.24 (0.95, 1.62)	0.113
BPAP	1.18 (0.84, 1.67)	0.341	1.45 (0.93, 2.29)	0.108
BPB	1.10 (0.62, 1.95)	0.746	1.42 (0.69, 2.93)	0.341
TBBPA	1.29 (1.01, 1.65)	0.045	1.46 (1.06, 2.05)	0.023
BPFL	1.29 (0.97, 1.73)	0.082	1.47 (1.01, 2.18)	0.047

Abbreviations: OR, odds ratio; CI, confidence interval. Unconditional logistic regression was used to compute ORs and CIs between bisphenols and precocious puberty. The models were adjusted for child age, child residence, child body mass index (BMI), guardian education, parity, mother’s BMI, sleep duration, and time spent in outdoor activities. *p* value < 0.05.

## Data Availability

Data will be made available upon reasonable request from the corresponding authors.

## Supplementary Materials

The following supporting information can be downloaded at: https://www.mdpi.com/article/10.3390/toxics11110905/s1, Figure S1: Flow chart of the study participants; Figure S2: Directed acyclic graph to select the minimal sufficient adjustment sets for estimating the total effect of bisphenols on precocious puberty; Figure S3: Pearson’s correlation coefficients test for 7 bisphenols with detection rates of greater than 49% in the (A) cases (N = 120) and (B) controls (N = 145). *p* < 0.05; Figure S4: Bivariate interactions between bisphenols and precocious puberty, when the single exposure-outcome function of the mixed bisphenols by second exposure of the mixed bisphenols was set at the percentiles 10th, 50th, and 90th percentiles, and compared with the remained bisphenols set at their median; Table S1: PIPs for group inclusion and conditional inclusion in the BKMR model (N = 120 cases and 145 controls); Table S2: Quantile-based g-computation of the mixture of bisphenols on precocious puberty and the relative contribution of each component in the mixture.
