# Supplementary material for: Association between Urinary BPA Substitutes and Precocious Puberty among Girls: A Single-Exposure and Mixed Exposure Approach from a Chinese Case—Control Study
Source: Toxics. 2023 Nov 6;11(11):905. doi: 10.3390/toxics11110905 (PMC10675366; doi:10.3390/toxics11110905)
Supplement: Supplementary file 1 [file toxics-11-00905-s001.zip › toxics-2681122-supplementary.pdf]

toxics MDPI

Supplementary Materials: Association between Urinary BPA Substitutes and Precocious Puberty among Girls: A Single-Exposure and Mixed Exposure Approach from a Chinese Case-Control Study

Francis Manyori Bigambo, Dandan Wang, Jian Sun, Xinliang Ding, Xiuzhu Li, Beibei Gao, Di Wu, Wei Gu, Mingzhi Zhang and Xu Wang

Table S1. PIPs for group inclusion and conditional inclusion in the BKMR model (N=120 cases and 145 controls).

Bisphenols	Group	Precocious puberty	
		**Group PIP**	**Conditional PIP**
BPA	1	0.88	**0.90**
BPB	1	0.88	0.10
BPS	2	0.74	0.34
BPAF	2	0.74	0.01
BPAP	2	0.74	0.05
TBBPA	2	0.74	**0.53**
BPFL	2	0.74	0.07

Abbreviations: PIPs, Posterior inclusion probabilities; BKMR, Bayesian kernel machine regression. The PIP was significant at the value ≥ 0.5.

Table S2. Quantile-based g-computation of the mixture of Bisphenols on precocious puberty and the relative contribution of each component in the mixture.

**Models**	**OR (95%CI)**	P-value	Weights								
			Direction	Effect	BPA	BPB	BPS	BPAF	BPAP	TBBPA	BPFL
Model 1	-0.10(-0.42, 0.22)	0.558	-	-	-	-	-	-	-	-	-
Model 2	0.20(-0.18, 0.59)	0.300	-	-	-	-	-	-	--	-	-
Model 3	0.12(-0.30, 0.53)	0.578	Pos	0.99		-	0.53	0.16	0.24	0.08	
			Neg	-0.87	0.30	0.34					0.36

Note: -, No expression. Model 1: Unadjusted. Model 2: Adjusted for child age, child BMI, mother BMI, guardian education, and child resident. Model 3: Child age, child resident, child body mass index (BMI), guardian education, parity, mother BMI, sleep duration, and time spent in outdoor activities.

Figure S1. Flow chart of the study participants.

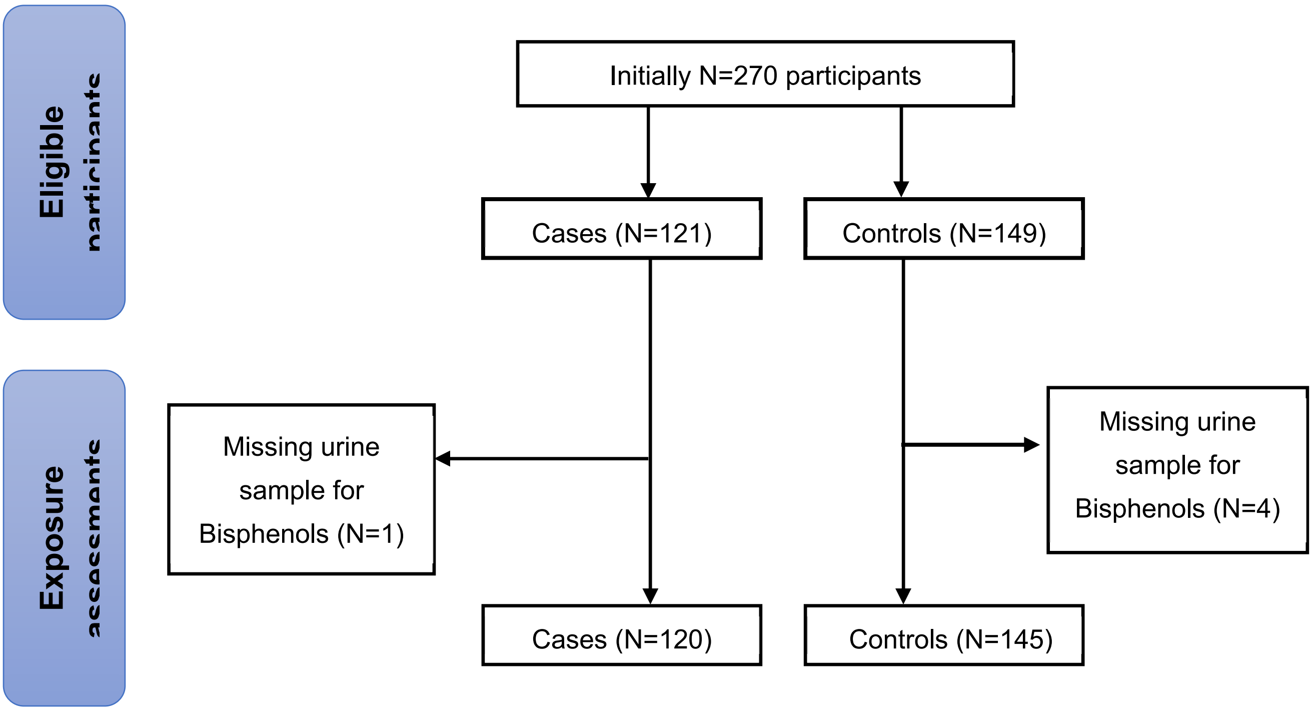

Figure S2. Directed acyclic graph to select the minimal sufficient adjustment sets for estimating the total effect of Bisphenols on precocious puberty.

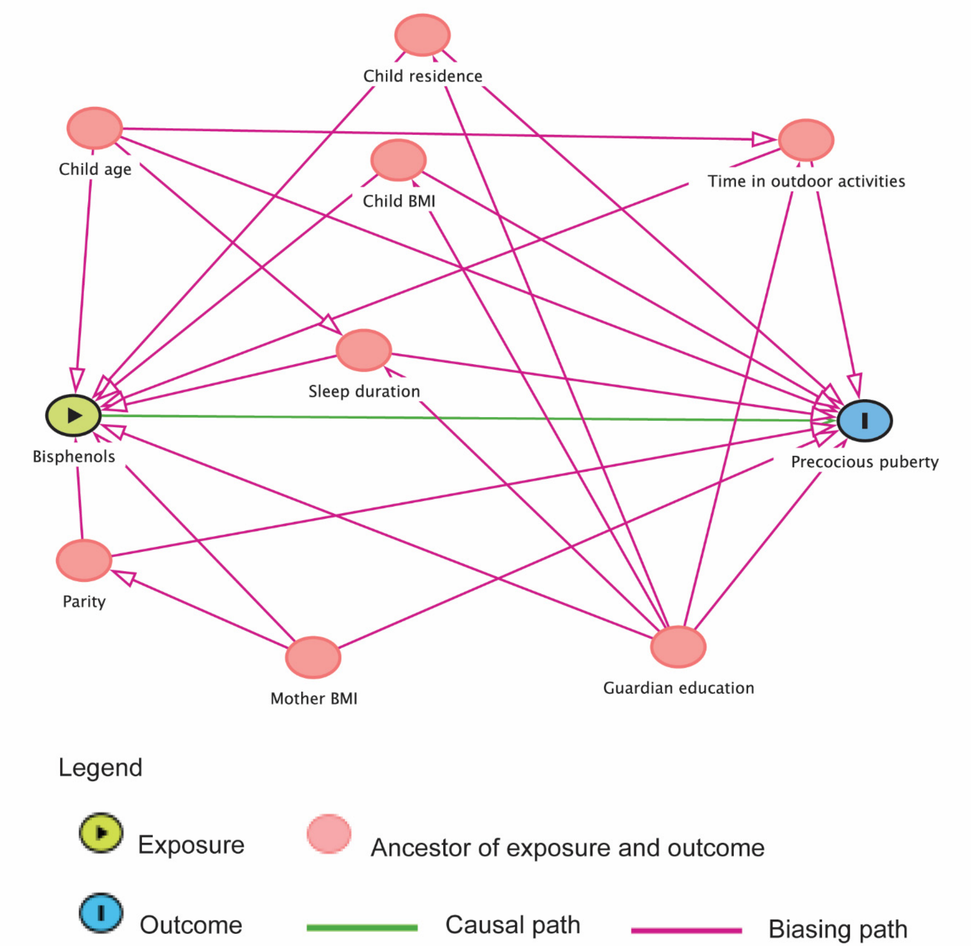

Figure S3. Pearson's correlation coefficients test for 7 Bisphenols with detection rates of greater than 49% in the (A) cases (N =120) and (B) controls (N = 145). *P< 0.05.*

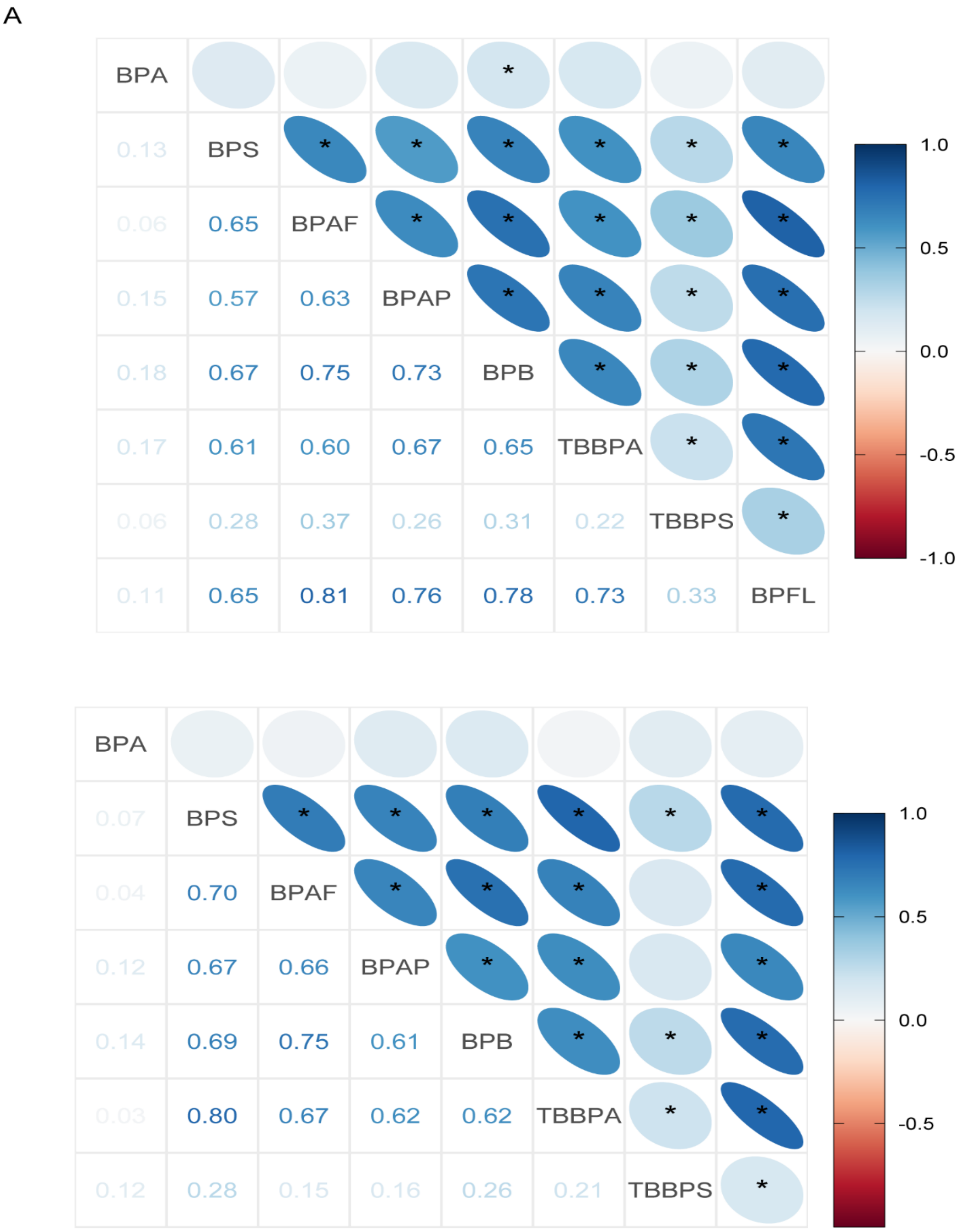

Figure S4. Bivariate interactions between Bisphenols and precocious puberty, when the single exposure-outcome function of the mixed Bisphenols by second exposure of the mixed Bisphenols was set at the percentiles 10th, 50th, and 90th percentiles, and compared with the remained Bisphenols set at their median.

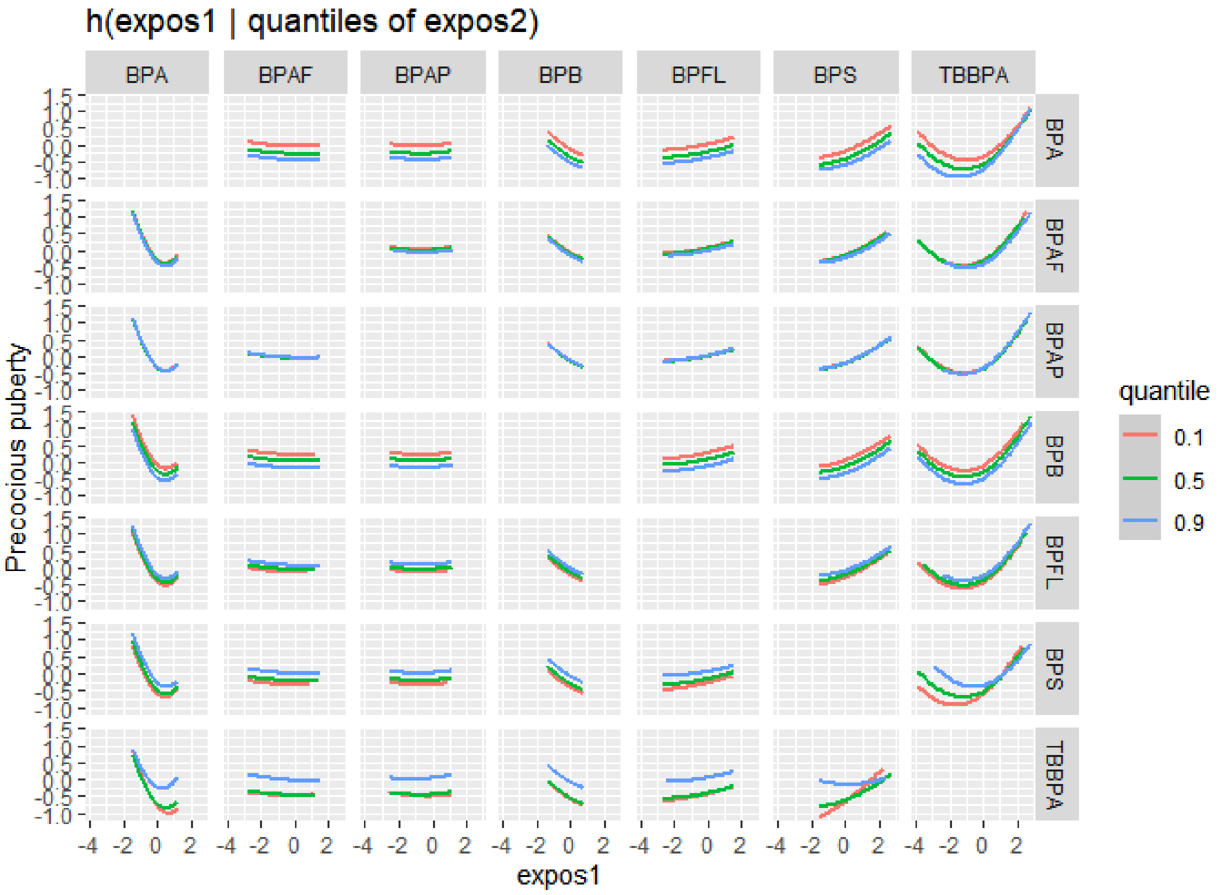

The Bayesian kernel machine regression was used to fit the models while adjusted for child age, child resident, child body mass index (BMI), guardian education, parity, mother BMI, sleep duration, and time spent in outdoor activities.
